# Isoprenoid-Derived Metabolites and Sugars in the Regulation of Flowering Time: Does Day Length Matter?

**DOI:** 10.3389/fpls.2021.765995

**Published:** 2021-12-24

**Authors:** Katarzyna Gawarecka, Ji Hoon Ahn

**Affiliations:** Department of Life Sciences, Korea University, Seoul, South Korea

**Keywords:** isoprenoid-derived metabolites, phytohormones, chlorophyll, flowering time, photoperiod, sugars

## Abstract

In plants, a diverse set of pathways regulate the transition to flowering, leading to remarkable developmental flexibility. Although the importance of photoperiod in the regulation of flowering time is well known, increasing evidence suggests the existence of crosstalk among the flowering pathways regulated by photoperiod and metabolic pathways. For example, isoprenoid-derived phytohormones (abscisic acid, gibberellins, brassinosteroids, and cytokinins) play important roles in regulating flowering time. Moreover, emerging evidence reveals that other metabolites, such as chlorophylls and carotenoids, as well as sugar metabolism and sugar accumulation, also affect flowering time. In this review, we summarize recent findings on the roles of isoprenoid-derived metabolites and sugars in the regulation of flowering time and how day length affects these factors.

## Introduction

Plants have a complex signaling network that adjusts flowering time in response to environmental conditions. Extensive studies have examined how this signaling network is regulated by environmental factors, such as day length (photoperiod) and temperature, and by genetic factors (Kinoshita and Richter, 2020; Renau-Morata et al., 2020; Susila et al., 2021b). Studies of the genetic factors regulating flowering have shown that flowering time genes (FTGs) include activators and repressors of flowering and the timing of flowering depends on the balance between these activities (Jin and Ahn, 2021). Among the genes involved in flowering activation are: *AGAMOUS-LIKE 24* (*AGL24*)*, CONSTANS* (*CO*), *FLOWERING CONTROL LOCUS A* (*FCA*), *FLOWERING LOCUS T* (*FT*), *GIGANTEA* (*GI*), *PHYTOCHROME A* (*PHYA*), *CRYPTOCHROME1* (*CRY1*)*, CRY2, SQUAMOSA PROMOTER BINDING PROTEIN-LIKE 3* (*SPL3*), *SPL9*, *SUPPRESSOR OF OVEREXPRESSION OF CONSTANS 1* (*SOC1*), and *TWIN SISTER OF FT* (*TSF*; Jaudal et al., 2020; Kim et al., 2020; Yu et al., 2020). The genes involved in repressing flowering include *AGL15*, *AGL18*, *CIRCADIAN CLOCK ASSOCIATED 1* (*CCA1*), *EARLY FLOWERING 3* (*ELF3*), *ELONGATED HYPOCOTYL* (*HY1*) and *HY2*, *FLOWERING LOCUS C* (*FLC*), *FLOWERING LOCUS M* (*FLM*), *LATE ELONGATED HYPOCOTYL* (*LHY*), *MADS AFFECTING FLOWERING 2* to *5* (*MAF2-5*), *PHYTOCHROME B* (*PHYB*), *SHORT VEGETATIVE PHASE* (*SVP*), *TIMING OF CAB EXPRESSION* (*TOC1*), and *ZEITLUPE* (*ZTL*; Airoldi et al., 2015; Yu et al., 2020; Zhao et al., 2021).

Among the environmental factors affecting flowering, scientists have known about the importance of the photoperiod for almost 100 years, since Garner and Allard (1922) showed that some plants cannot flower unless they experience a certain day length. Based on their flowering responses to different photoperiods, three groups of plants have been established: short-day (SD), long-day (LD), and day-neutral plants (Kinoshita and Richter, 2020). In these plants, the introduction of a different day-to-night ratio results in changes in the expression of FTGs and subsequent signal transmission, which eventually affects flowering time. Experiments using *Arabidopsis thaliana* revealed that *FT*, *GI*, *CO*, *SOC1*, *CCA1*, *CONSTITUTIVELY PHOTOMORPHOGENIC 1* (*COP1*), *CYCLING DOF FACTORs* (*CDFs*), *HIGH EXPRESSION OF OSMOTICALLY RESPONSIVE GENES 1* (*HOS1*), *ADAGIO1* (*ADO1*)*/ZTL*, *AGL24*, *FLAVIN-BINDING KELCH REPEAT F-BOX* (*FKF1*), *PHYA*, *PHYB*, and *CRYs* participate in the response to the day-to-night ratio in modulating flowering time (Cao et al., 2021).

Plants have sophisticated signaling networks that mediate their light responses. Perception of light of different wavelengths by phytochromes, cryptochromes, and FKF1 triggers a signaling cascade (Oakenfull and Davis, 2017). In this cascade, signals from different light conditions lead to expression of the direct targets of photoreceptors, such as *CCA1*, *LHY*, *COP1*, *HOS1*, *CDF*, and *ADO1*/*ZTL* (Golembeski et al., 2014), thereby influencing the expression of downstream targets in the photoperiod pathway (e.g., *GI* and *CO*). In light signaling, CO stability is important for activation of the floral transition. For instance, the E3 ligases COP1 and HOS1 interact to regulate CO abundance (Lazaro et al., 2012). In the night, COP1 interacts with SUPPRESSSOR OF PHY A-105 (SPA) to regulate CO stability (Jang et al., 2008; Kinoshita and Richter, 2020).

In addition to photoperiod, a diverse group of environmental cues affect the flowering signaling network. For instance, nutrient availability affects flowering time, such that low nitrogen concentration accelerates flowering. Low nitrogen prevents phosphorylation of FLOWERING BHLH4 (FBH4) and promotes its nuclear localization (Sanagi et al., 2021). FBH4 binds to the *CO* promoter and enhances transcription of *CO* and its downstream genes that act in the photoperiod pathway. Thus, under low nitrogen conditions, flowering is accelerated due to increased expression of genes acting in the photoperiod pathway. In addition, studies of nitrate transporters showed that LD photoperiod improves nitrogen uptake and positively regulates flowering time (Ye et al., 2021).

Salt stress has a strong effect on flowering time. Results from Arabidopsis (Kim et al., 2007; Li et al., 2007; Ma et al., 2015; Osnato et al., 2021), rice (Sarhadi et al., 2012; Batlang et al., 2013; Wang et al., 2021b), soybean (*Glycine max*; Cheng et al., 2020; Otie et al., 2021), and barley (*Hordeum vulgare*; Agarwal et al., 2019; Wiegmann et al., 2019) showed that plants exposed to salt stress flowered late. For instance, in Arabidopsis, PHOSPHATIDYLINOSITOL 4-KINASE*γ*3 (PI4K*γ*3) accumulates when plants are exposed to salt stress. PI4K*γ*3 positively regulates *FLC* expression and negatively regulates *GI*, *FT*, and *SOC1* expression, thus delaying flowering (Akhter et al., 2016). Indeed, *PI4Kγ3*-overexpressing lines showed a late-flowering phenotype as well as higher salt tolerance, whereas *pi4k* mutants showed opposite phenotypes.

Drought stress also affects the timing of flowering. Plants exposed to drought stress respond by flowering earlier (known as drought escape) or by acclimating and delaying flowering until the conditions change (known as drought tolerance; Shavrukov et al., 2017). Interestingly, photoperiod affects drought stress responses, such that Arabidopsis plants exposed to drought stress under LD conditions flowered earlier, but Arabidopsis plants exposed to the same stress under SD conditions flowered later (Riboni et al., 2013). These findings demonstrate that plants respond differently to environmental conditions when they are exposed to different day lengths.

In recent years, increasing evidence has shown that signals from isoprenoid-derived compounds, such as phytohormones [gibberellins (GBs), abscisic acid (ABA), brassinolides, and cytokinins (CKs)] and photosynthetic pigments (chlorophylls and carotenoids), as well as metabolites originating from photosynthesis (sucrose and trehalose-6-phosphate), affect flowering time when plants are exposed to SD or LD conditions. In this review, we focus on findings from the last 5 years and summarize the role of isoprenoid-derived metabolites and sugars in the regulation of flowering time and how day length affects signaling from these metabolites.

## Isoprenoid-Derived Metabolites in Flowering Time Regulation and the Effect of Photoperiod

Isoprenoids (terpenes) are a very large, diverse group of metabolites present in all living organisms (Swiezewska and Danikiewicz, 2005). Plant isoprenoids include primary and secondary metabolites involved in photosynthesis (chlorophylls, carotenoids, and plastoquinone), modulation of membrane properties (phytosterols, polyprenols, and dolichols), growth/development [gibberellins, brassinosteroids (BRs), and cytokinins], and plant defenses against biotic and abiotic stress (ABA; Boncan et al., 2020).

**Figure 1:**
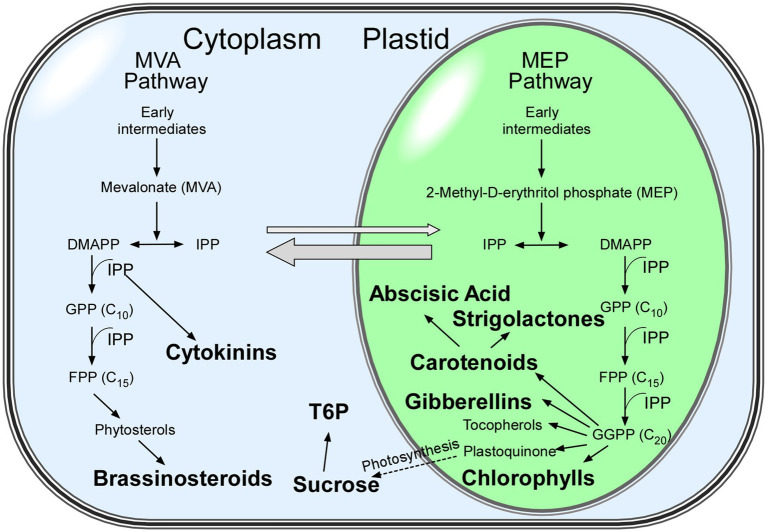
Isoprenoid biosynthetic pathways in plants. Metabolites discussed in this review are shown in bold. The mevalonate (MVA) and methylerythritol phosphate (MEP) pathways both generate isopentenyl diphosphate (IPP) in parallel and contribute to particular isoprenoids (Swiezewska and Danikiewicz, 2005). Thick grey arrows show the exchange of intermediates between the MVA and MEP pathways. Abbreviations: DMAPP: dimethylallyl diphosphate; FPP: farnesyl diphosphate; GPP: geranyl diphosphate; GGPP: geranylgeranyl diphosphate; IPP: isopentenyl diphosphate.

Plants have two isoprenoid biosynthetic pathways, the mevalonate (MVA) pathway in the cytoplasm, which is responsible for the biosynthesis of sterols and plant hormones, such as cytokinins and brassinosteroids, and the methylerythritol phosphate (MEP) pathway in plastids, which is responsible for the biosynthesis of components involved in photosynthesis (chlorophylls, carotenoids, and plastoquinone) and phytohormones (gibberellins and abscisic acid; Swiezewska and Danikiewicz, 2005; Figure 1). Many isoprenoid-derived compounds are involved in flowering time and their effects can be modulated by day length. In the following sections, we discuss how the signals from isoprenoid-derived phytohormones and photosynthetic pigments affect flowering time in response to different photoperiods and light conditions.

### Gibberellins

Gibberellins (GAs) are diterpene phytohormones that are produced from the plastid pool of isopentenyl diphosphate. So far, 136 molecularly distinct forms of gibberellins (GA_1_–GA_136_) have been identified in plants, fungi, and bacteria (Tudzynski et al., 2016). Among these GAs, GA_1_, GA_3_, GA_4_, and GA_7_ are the major bioactive forms (Yamaguchi, 2008) that are known to regulate a number of developmental processes in plants, including the floral transition (Yamaguchi, 2008; Gupta and Chakrabarty, 2013). The effect of GAs on flowering is species-specific; for instance, in *Arabidopsis thaliana*, GAs induces flowering under non-inductive photoperiodic conditions (Eriksson et al., 2006; Yamaguchi, 2008), whereas they repress flowering in several woody plant species, including apple (*Malus* spp.; Bertelsen and Tustin, 2002), citrus (*Citrus* spp.; Goldberg-Moeller et al., 2013), grapevine (*Vitis vinifera*; Boss and Thomas, 2002), and peach (*Prunus persica*; Southwick et al., 1995).

Levels of GAs are directly linked to flowering time (Eriksson et al., 2006; Bao et al., 2020). For example, classical experiments on *Lolium temulentum* showed that exogenous application of GAs was functionally equivalent to a single LD treatment in triggering flowering (Pharis et al., 1987). GA levels decrease if the GA biosynthesis gene *GA20-OXIDASE2* (*GA20ox2*) is not activated or if the GA catabolism gene *GA2ox7* is overexpressed; both conditions result in late flowering, due to reduced *FT* mRNA levels under LD conditions (Hisamatsu and King, 2008; Porri et al., 2012) and low expression levels of *SOC1* and *LFY* under SD conditions (Blázquez et al., 1998; Moon et al., 2003). *FACKEL* (*FK*), which encodes a protein involved in sterol synthesis, may affect GA accumulation (Huang et al., 2017). The *fk* mutants showed late flowering due to the elevated levels of *FLC*, together with the altered mRNA levels of GA metabolism genes (leading to reduced levels of endogenous GAs). Furthermore, vernalization (which represses expression of the floral inhibitor *FLC*) and application of exogenous GA_3_ rescued the late-flowering phenotype of *fk* mutants under LD conditions (Huang et al., 2017), suggesting that *FK* is important for crosstalk between the GA and vernalization pathways.

The modification of GAs also affects flowering time by modulating ratios of biologically active and inactive GAs. For example, hydroxylation of carbon 13 of GA molecules deactivates GA and thus can delay flowering (He et al., 2019). Overexpression of *CYP72A9* (encoding GA 13-hydroxylase) in Arabidopsis leads to the accumulation of inactive forms of GA under LD conditions and results in late flowering (He et al., 2019), suggesting that the ratio of inactive 13-OH and active 13-H GAs is important for the timing of the floral transition. These findings suggest that besides the overall GA levels, the ratio of biologically active and inactive forms of GA is also important for flowering time.

In Arabidopsis, the effect of GA on floral induction is much stronger under non-inductive photoperiodic conditions than under inductive conditions. Under SD (non-inductive) conditions, when *CO* transcript levels are low, GA independently regulates transcription of *SOC1*, *LFY*, *FRUITFULL* (*FUL*), and *SPLs* in the shoot apical meristem (SAM), which leads to induction of the floral transition (Eriksson et al., 2006; Jung et al., 2012; Andrés et al., 2014). Studies of the basic helix-loop-helix (bHLH) transcription factor gene *NO FLOWERING IN SHORT DAY* (*NFL*) also showed the importance of GA for flowering under SD conditions (Sharma et al., 2016). In *nfl* mutants, genes encoding enzymes responsible for GA degradation are upregulated. The *nfl* mutants fail to flower under SD conditions unless exogeneous GA is provided, implying that NFL is a key factor regulating the floral transition in the GA pathway under SD conditions. However, the precise molecular mechanism explaining NFL function awaits further investigation.

The floral repressor SVP also affects GA-mediated regulation of flowering in Arabidopsis. In the dark, SVP reduces GA biosynthesis *via* transcriptional repression of *GA20ox2*, which results in delayed flowering (Andrés et al., 2014). PHOSPORYLETHANOLAMINE CYTIDYLTRANSFERASE1 (PECT1) modulates the ratio of phosphatidylethanolamine:phosphatidylcholine (Mizoi et al., 2006). The artificial microRNA-mediated knockdown of *PECT1* in the SAM (*pFD::amiR-PECT1*) resulted in reduced *SVP* mRNA levels and consequent upregulation of *GA20ox2* in the SAM, leading to early flowering independent of the photoperiod (Susila et al., 2021a). These findings showed the importance of GAs in promoting the floral transition in plants with altered ratios of structural phospholipids (including phosphatidylethanolamine and phosphatidylcholine) and the role of *SVP*, which provides a link between altered phospholipid ratios and GA biosynthesis. However, the underlying mechanism of how these structural phospholipids affect *SVP* transcription remains elusive.

DELLA proteins, which are negative regulators of GA signaling, participate in many developmental changes in plants, including flowering transition (Tyler et al., 2004; Thomas et al., 2016). Arabidopsis plants have five genes encoding DELLA proteins: *GIBBERRELIN INSENSITIVE* (*GAI*), *REPRESSOR OF ga1-3* (*RGA*), *RGA-like 1* (*RGL1*), *RGL2*, and *RGL3* (Itoh et al., 2008; Sun, 2011; Davière and Achard, 2013; Locascio et al., 2013). These DELLA proteins form complexes with various factors that affect flowering time and regulate the expression of FTGs. For example, regulation of *FT* expression by CO under LD conditions depends on GA status (Wang et al., 2016). When GA levels are low, DELLA proteins form a complex with CO and prevent it from binding to the *FT* promoter, leading to reduced *FT* expression and hence delayed flowering. The DELLA-CO protein-protein interaction also inhibits the formation of the floral-inducing CO-NUCLEAR FACTOR Y SUBUNIT B2 (NF-YB2) complex (Xu et al., 2016), which is required for the CO-mediated induction of *FT* and *SOC1* (Cao et al., 2014).

DELLA proteins interfere with the transcriptional activity of bHLH transcription factors by direct protein-protein interactions to modulate flowering time specifically under SD (Sharma et al., 2016) or LD conditions (Li et al., 2017). NFL encodes a bHLH family transcription factor and the non-flowering phenotype of *nfl* mutants, which is observed only under SD conditions, was rescued by the genetic inactivation of DELLAs (Sharma et al., 2016), suggesting that NFL regulates the floral transition primarily *via* the GA pathway under non-inductive photoperiodic conditions. Unlike NFL, the bHLH transcription factors bHLH48 and bHLH60 regulate flowering under LD conditions only, *via* direct regulation of *FT* transcription (Li et al., 2017). Loss of function of *bHLH48* and *bHLH60* resulted in late flowering, whereas their overexpression led to early flowering under LD conditions. The DELLA protein RGL1 interacts with both bHLH48 and bHLH60 and the RGL1-bHLH48 interaction may reduce the binding of bHLH48 to the *FT* promoter, as exogenous GA_3_ promoted binding of bHLH48 to the *FT* promoter and hence accelerated flowering (Li et al., 2017), which is likely caused by triggering degradation of DELLA protein(s).

DELLA proteins also affect flowering time under LD conditions by interacting with WRKY DNA-BINDING PROTEIN 75 (WRKY 75; Zhang et al., 2018). WKRY75 functions in a FT-dependent manner, as *wrky75* mutants and *WRKY75*-overexpressing lines showed late and early flowering phenotypes, respectively, which were associated with changes in *FT* expression levels. Additionally, RGL1 and GAI physically interact with WRKY75 and suppress its transcriptional activation ability; GAs are necessary for releasing WRKY75 from its DELLA complexes and thus inducing *FT* transcription (Zhang et al., 2018). Furthermore, interaction of DELLA proteins with two functionally antagonistic WRKY transcription factors, WRKY12 (floral promoter) and WRKY13 (floral repressor), interfered with their ability to regulate *FUL* expression (Li et al., 2016c). WRKY12 positively regulates *FUL* expression, whereas WRKY13 represses it. Li et al. (2016c) hypothesized that homeostasis with more WRKY12 and less WRKY13 could promote GA-induced DELLA degradation and induce the floral transition. However, this hypothesis needs to be validated experimentally and the question of how this homeostasis promotes GA-mediated DELLA repression needs to be answered. Interestingly, DELLA proteins also interact with FLC, increasing the ability of FLC to repress its downstream targets, primarily *SOC1*, and thus leading to late flowering (Li et al., 2016b). Application of exogenous GA accelerated flowering of FLC-overexpressing lines under both LD and SD conditions, most likely by inhibiting DELLA-FLC interactions that lead to reduced repression of its targets by FLC (Li et al., 2016b).

Degradation of DELLA proteins is a key mechanism for regulating their activity and the regulation of GA responses in response to light provides an interesting example of this regulation. For instance, in response to blue light, the major blue-light photoreceptor CRY1 interacts with the GA receptor GA-INSENSITIVE DWARF1 (GID1) and inhibits the association between GID1 and DELLAs, eventually leading to the inhibition of GA signaling (Zhong et al., 2021). In the presence of GAs, DELLA proteins are actively ubiquitinated and FLAVIN-BINDING KELCH REPEAT F-BOX1 (FKF1) plays a role in this ubiquitination process under LD conditions (Yan et al., 2020). Plants that lack *FKF1* accumulated more DELLA proteins; thus, they were less sensitive to GA treatment and showed a late-flowering phenotype under LD conditions (Yan et al., 2020).

The transcription factor MYC3 participates in GA regulation under SD conditions. Under non-inductive conditions, MYC3 is stabilized by its interactions with DELLAs, and the resulting stabilized DELLAs-MYC3 complexes outcompete CO in binding to the *FT* promoter and hence repress *FT* transcription. Under inductive conditions, GA modulates MYC3 protein abundance by promoting degradation of DELLAs and hence accelerated flowering (Bao et al., 2019).

DELLA proteins negatively regulate GA biosynthesis and GA-ASSOCIATED FACTOR 1 (GAF1) participates in that regulation (Fukazawa et al., 2017). DELLA proteins form a complex with GAF1 during GA deficiency and promote GA biosynthesis by directly binding to the *GA20ox2* promoter. Higher levels of GA promote DELLA degradation and destabilize the DELLA-GAF1 complex, which leads to repression of *GA20ox2* and inhibition of GA biosynthesis. Recently, Fukazawa et al. (2021) revealed that GAF1 forms a transcriptional repressor complex with TOPLESS-RELATED (TPR) and upregulates the expression of *FT* and *SOC1* by repressing the expression of *EARLY FLOWERING3* (*ELF3*), *SVP*, *TEMPRANILLO1* (*TEM1*), and *TEM2*. The GA-dependent regulation by the GAF1-TPR complex occurs in a tissue-specific manner, such that in the leaf, the GAF1-TPR complex represses the expression of *ELF3*, *TEMs*, and *SVP* to promote *FT* expression, whereas in the SAM, the GAF1-TPR complex represses the expression of *SVP* to promote *SOC1* expression (Fukazawa et al., 2021).

Under SD conditions, SPL15 and SOC1 function together to promote flowering by direct activation of miR172b and *FUL* in the SAM; DELLA proteins also interact with SPL15 (Hyun et al., 2016). These findings showed that GA has a positive role in flowering induction under SD conditions, as GA-induced degradation of DELLAs releases SPL15 from the SPL15-DELLA complex. Additionally, DELLA proteins are proposed to be involved in the regulation of light-sensing signaling, which affects flowering time under LD conditions (Feng et al., 2008; Li et al., 2016a). DELLAs inhibit PHYTOCHROME-INTERACTING FACTOR1 (PIF1) and PIF3, 4, and 5, key regulators of light-regulated plant development, by sequestering their DNA-recognition domains (Feng et al., 2008; Li et al., 2016a). Similarly, maize ZmPIF4 and ZmPIF5 interact with Arabidopsis DELLA protein (RGA) and their heterologous overexpression resulted in early flowering in Arabidopsis (Shi et al., 2018), suggesting that this regulatory mechanism is conserved across plant species.

In addition to DELLA proteins, several other players also regulate GA signaling during the floral transition. For example, PICKLE (PKL) may function antagonistically to DELLA proteins, as the *pkl* mutation suppressed the early flowering phenotype of *della* pentuple mutants under LD conditions. The *pkl gai-1* double mutants flowered later than *gai-1* single mutants (Park et al., 2017), revealing that the GA-mediated regulation of flowering requires PKL activity. In addition, carbohydrates are important for GA signaling, as low starch accumulation during the night as a result of insufficient photosynthesis can inhibit GA synthesis by downregulating *GA3ox1* (Prasetyaningrum et al., 2021).

**Figure 2:**
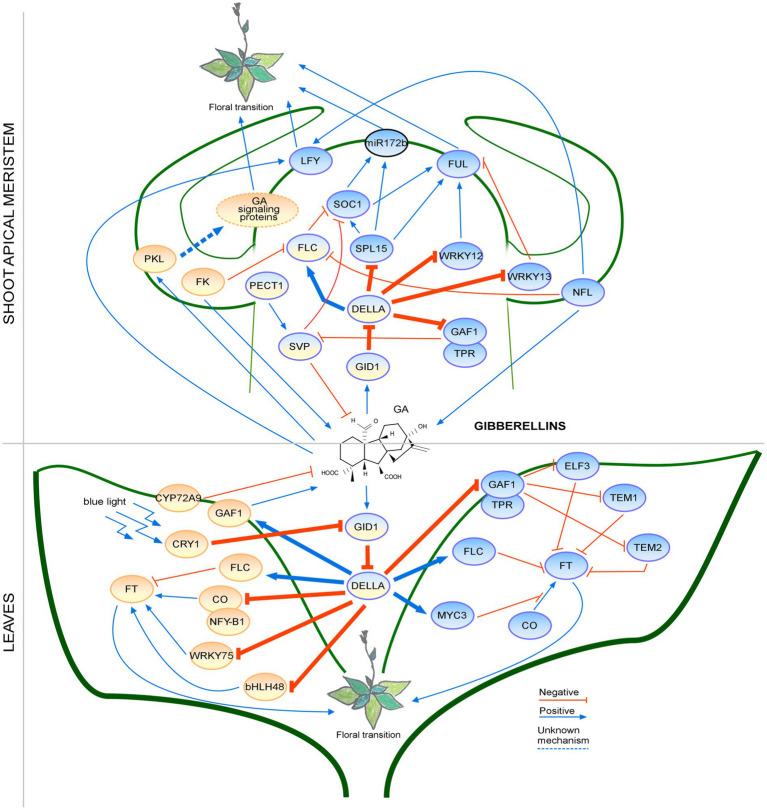
Regulation of the floral transition by the gibberellin signaling pathway in different photoperiods in SAM and Leaves. Proteins involved in signaling pathways under SD, LD or both photoperiods are shown in ovals with a blue, orange or blue-orange background, respectively. Positive regulatory interactions are depicted by blue arrows and negative interactions are depicted by red T-bars. The thick lines represent protein-protein interactions, whereas thin lines indicate transcriptional regulation. Unknown mechanisms are depicted by dotted lines. Protein complexes are depicted as partially overlapping ovals. The regulation by the PKL is not fully understood; therefore, the GA signaling proteins are placed in an oval with a dotted border. miRNA is indicated by an oval with a black border.

These findings highlight the complexity of GA signaling pathways and show the connection between GA signaling and photoperiod in the regulation of flowering time (Figure 2). Emerging research has identified factors that regulate flowering by interacting with DELLAs and are activated by GA, revealing the interconnections among different regulatory pathways. Further investigation is needed to elucidate how the GA signaling pathway connects with responses to other environmental cues.

### Brassinosteroids

Brassinosteroids (BRs) are steroid phytohormones biosynthesized from cholesterol, campesterol, and *β*-sitosterol through the cytosolic MVA pathway (Bajguz et al., 2020). Brassinolide (BL) is the most active BR; castasterone and typhasterol may also function in plant development (Yokota, 1997). Temperature and light regulate BR biosynthesis and BRs are involved in several developmental processes, including flowering time (Domagalska et al., 2010; Ye et al., 2010; Jiang et al., 2013; Nolan et al., 2020). Although the BR biosynthesis pathway in plants is well understood, only a few mutants with impaired BR accumulation or signaling have been characterized in the context of flowering time.

BRASSINOSTEROID INSENSITIVE 1 (BRI1) acts as a BR sensor; binding of BRs to the extracellular domain of BRI1 activates its kinase activity. BRI1-ASSOCIATED RECEPTOR KINASE1 (BAK1) is also recruited during BRI1 activation. Through a series of steps, activated BRI1 then activates the transcription factors BRASSINAZOLE-RESISTANT 1 (BZR1) and BR1-EMS-SUPPRESSOR 1 (BES1) to initiate the transcriptional reprogramming of their downstream genes (Zhu et al., 2013; Bajguz et al., 2020). In the context of flowering time, BRs have been reported to both repress and promote flowering (Domagalska et al., 2007; Li et al., 2018). Loss of *BRI1* function in the Wassilewskija (Ws) background results in delayed flowering due to elevated *FLC* expression (Domagalska et al., 2007). By contrast, loss of *BRI1* function in the Colombia (Col-0) background results in accelerated flowering (Li et al., 2018). In Arabidopsis, mutants with low BR levels, such as *constitutive photomorphogenic and dwarf* (*cpd*), *dwarf 4* (*dwf4*), or *de-etiolated 2* (*det2*), showed very weak late flowering under LD conditions and did not bolt under SD conditions (Chory et al., 1991; Domagalska et al., 2007, 2010), possibly due to their severe developmental defects, suggesting that photoperiod affects BR signaling. However, Domagalska et al. (2010) found that overexpression of *DWF4* in Arabidopsis did not affect flowering time under both LD and SD conditions, suggesting that overexpression of a single enzyme might not be sufficient to increase BR levels, as the authors did not quantify the BR levels in transgenic plants. Another possibility is that BRs do not exert a strong effect on flowering time and the inability of BR-deficient mutants to flower might be due to their severe developmental defects. Therefore, further experiments are required to determine whether genetic uncoupling of the other developmental defects from floral transition can affect the non-flowering phenotype of these BR-defective mutants under non-inductive photoperiodic conditions.

The BR signaling-mediated flowering pathway is conserved among flowering plants, as heterologous overexpression of a wheat (*Triticum aestivum*) gene encoding BRI (*TaBRI1*) in Arabidopsis induced early flowering (Singh et al., 2016). Similarly, heterologous overexpression of the soybean BR biosynthesis gene *GmCPD*, encoding an enzyme responsible for the hydroxylation of carbon 23 in BRs, in Arabidopsis *cpd* mutants rescued the developmental defects of *cpd* mutants, including late flowering (Wang et al., 2015a). Additionally, photoperiod regulates *GmCPD* expression in soybean and soybean plants with high *CPD* levels showed a photoperiod-dependent flowering phenotype. Analyses of FTG expression showed that the observed flowering time phenotype cannot be explained by *GmFT* expression levels, which suggests the involvement of additional players. Hence further research is required to decipher the underlying mechanism by which CPD modulates flowering.

BR autoregulates its own biosynthesis. PIFs are involved in this autoregulation and promote BR signaling during the floral transition (Martínez et al., 2018). PIFs positively regulate BR biosynthesis by interacting with the BR-responsive transcription factor BES1 and promoting BR signaling in response to circadian rhythms. The balance between BES1 and PIF4 levels defines whether BES1 acts as a repressor or an activator of BR biosynthesis genes (Martínez et al., 2018). If *PIF4* expression is reduced, BES1 proteins form homodimers and repress BR biosynthesis, which diminishes the BR response, whereas the accumulation of PIF4 increases BR levels by competing for BES1 homodimerization (Martínez et al., 2018). In Arabidopsis, salinity (NaCl) and ABA suppress PIF4 function and BR accumulation most likely by inhibiting the PIF-BES1 signaling module in a light-dependent manner (Hayes et al., 2019).

A recent study revealed that BRs affect photoperiodic flowering (Wang et al., 2019). The BR-activated BES1 transcription factor directly binds to the BR ENHANCED EXPRESSION 1 (BEE1) promoter region and induces its transcription, and BEE1 in turn directly induces *FT* transcription and hence promotes flowering (Wang et al., 2019). CRY2 physically interacts with BEE1 in response to blue light and enhances its DNA-binding ability to further increase its transcriptional activity. BEE1 accumulates when plants are moved from dark or red light to blue light; however, BEE1 is degraded when plants are moved to the dark, suggesting that BEE1 protein is stabilized by blue light independent of CRY2. Overexpression of *BEE1* partially rescued the late-flowering phenotype of *cry1 cry2* double mutants (Wang et al., 2019), which suggested an additional FT- and BR-dependent mechanism(s) regulating flowering in the photoperiod pathway.

BRs interact with GAs to regulate plant development and flowering (Unterholzner et al., 2015). For example, overexpression of the GA biosynthesis gene *GA20ox1* in a BR signaling mutant (*bri1*) rescued the late-flowering phenotype. However, it seems that these pathways may work together only partially, as the exogenous application of GA_4_ and complementation using the *BRI1* promoter-driven *GA20ox1* partially rescued the flowering phenotype of *bri1* mutants (Unterholzner et al., 2015).

The effect of BR on flowering was also seen in plant species other than Arabidopsis. For example, a longer vegetative phase was observed in tobacco (*Nicotiana tabacum*) plants overexpressing the BR biosynthesis gene *PcDWF1* from pear (*Pyrus communis*); biochemical analyses confirmed that the transgenic plants had higher accumulation of BR (Zheng et al., 2020). A similar effect of BRs was observed in wheat, such that exogenous application of BR (24-epibrassinolide) negatively affected flowering in wheat, whereas chemical inhibition of BR biosynthesis with brassinazole promoted flowering (Janeczko et al., 2015).

**Figure 3:**
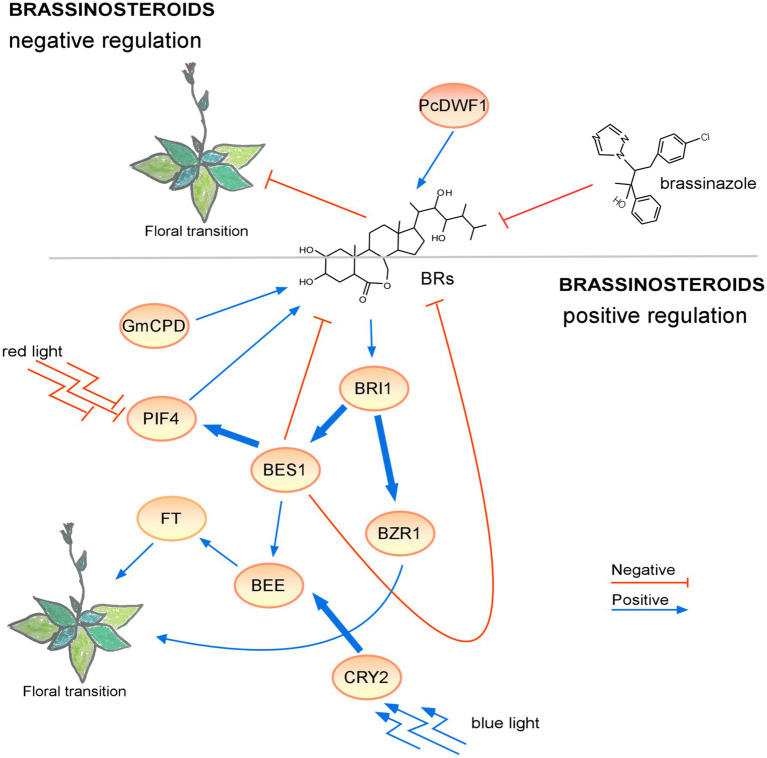
Regulation of the floral transition by the brassinosteroid signaling pathway in different photoperiods. Proteins involved in positive or negative signaling pathways under LD are shown in ovals with a light or dark orange background, respectively. Under short-day conditions, the lack of brassinosteroids resulted in a non-flowering phenotype. The positive regulatory interactions are depicted by blue arrows and negative interactions are depicted by red T-bars. The thick lines represent protein-protein interactions, whereas thin lines indicate transcriptional regulation. Protein complexes are depicted as partially overlapping ovals.

These findings showed that newly identified genes involved in BR metabolism and signaling affect flowering time and BRs may have dual effects on flowering (Figure 3). These observations imply that the topic of BRs as regulators of the floral transition is very complex and ripe for further investigation. Additional experiments will likely shed some light on the mechanisms of BR signaling during the floral transition.

### Abscisic Acid

The phytohormone ABA is synthesized from carotenoids in plastids and is involved in plant development and stress responses, which affect flowering time. ABA accumulation is detected by the ABA sensors PYRABACTIN RESISTANCE 1 (PYR1) and PYR1-like (PYL), which transduce signals by inhibiting PP2C phosphatases. PP2C activates SUCROSE-NON-FERMENTING (SNF1)-related protein 2 (SnRK2) and induces ABA-related responses, which can be modulated by light and photoperiod (Yadukrishnan and Datta, 2020). SnRK2 phosphorylates its target proteins, including the bZIP transcription factors ABA-RESPONSIVE ELEMENT (ABRE)-BINDING FACTORs (ABF1, ABF2, ABF3, and ABF4) and ABSCISIC ACID INSENSITIVE4 (ABI4) and ABI5. Mutations in the *ABF* or *ABI* genes altered flowering time (Wang et al., 2013; Sugimoto et al., 2014; Yoshida et al., 2014; Riboni et al., 2016; Shu et al., 2016; Li et al., 2019a). In addition, a recent study showed that upregulation of *ABF2*, together with repression of the expression of ABA receptor genes and *LFY*, was induced by the formation of the TERMINAL FLOWER1 (TFL1)-FD complex under SD conditions (Zhu et al., 2020).

Arabidopsis *fd* and *fd* paralog (*fdp*) mutants, which showed late and early flowering phenotypes, under LD conditions, respectively, and FD and FDP directly bind to the *LFY* and *AP1* sequences. It has been reported, that FD and FDP also affect the expression of ABA signaling-related genes (*ABI5* and *ABF3*; Romera-Branchat et al., 2020). The *fd* and *fdp* mutants showed different flowering time phenotypes, but the influence of ABA in mutants that impair the ABA signaling pathway was not examined. It would be interesting to investigate whether the LD-dependent floral transition of *fd* and *fdp* mutants depends on ABA.

Abscisic acid regulates flowering time in both positive and negative ways (Yoshida et al., 2014; Shu et al., 2016). To acclimate to new environmental conditions, plants modulate their response to ABA and modify the expression of FTGs. For example, ABFs modulate *CO* expression to control flowering time, as Arabidopsis *areb1 areb2 abf1 abf3* quadruple mutants showed a late-flowering phenotype with reduced *CO* expression levels under LD conditions (Yoshida et al., 2014). Additionally, the overexpression of *ABI5-BINDING PROTEIN 2* (*AFP2*), a negative regulator of ABA signaling, resulted in downregulation of *CO* (Chang et al., 2019). Plants overexpressing *AFP2* showed a late-flowering phenotype under LD conditions; however, *afp2* mutants showed a weak early flowering phenotype with high *CO* expression levels. AFP2 forms a complex with CO and TOPLESS-RELATED PROTEIN2 to suppress transcriptional activity of CO, while AFP2 also mediates CO degradation during the night (Chang et al., 2019). In addition, ABFs affect *SOC1* expression levels and ABF3 and ABF4 play a role in this process regulating flowering time under LD conditions, specifically at 23°C (Hwang et al., 2019). Hwang et al. (2019) showed that the *abf2 abf3 abf4* triple mutants showed stronger late flowering than each single mutant and the late flowering was caused by the suppression of *SOC1* expression. ABF3 and ABF4 interact with NF-Y subunit C 3/4/9 to promote flowering by inducing *SOC1* transcription under drought conditions (Hwang et al., 2019). It is thus likely that Arabidopsis uses the ABF-NF-Y complex-SOC1 module to accelerate flowering and thus escape from drought stress conditions. These reports showed that the ABA positively regulates flowering time by the stabilization of CO or upregulation of *SOC1*.

In contrast, other studies showed that ABA may also have a negative effect on flowering time. Shu et al. (2016) reported that ABA negatively regulates flowering by upregulating the expression of *FLC*, which is a potent repressor of flowering. They showed that a lesion in *ABI4*, which is a close homolog of *ABI5* and plays a role in the ABA signaling network, causes an early flowering phenotype and *ABI4*-overexpressing plants show a late-flowering phenotype under SD and LD conditions. The flowering time change is attributed to the direct binding of ABI4 to the *FLC* promoter and activation of *FLC* expression (Shu et al., 2016). The negative regulation by ABA was also observed in plants overexpressing *ETHYLENE RESPONSE FACTOR 96* (*ERF96*), a positive regulator of the ABA response (Wang et al., 2015b). *ERF96*-overexpressing plants showed late flowering, together with the typical responses caused by elevated levels of ABA (i.e., reduced stomatal aperture and slow water loss; Wang et al., 2015b). Delayed flowering time, together with high tolerance to drought stress, was also observed in transgenic plants overexpressing the *MYB37* transcription factor gene (Yu et al., 2015). ABA’s negative effect was also described in transgenic cotton (*Gossypium hirsutum*) plants heterologously overexpressing Arabidopsis *RELATED TO ABA-INSENSITIVE3/VIVIPAROUS1 (RAV1)* or *RAV2*; *RAV1(2)-*overexpressing plants showed a late-flowering phenotype under both normal and drought stress conditions under LD conditions (Mittal et al., 2015). The RAV1 was also reported to be a target of SnRK2 kinases (Feng et al., 2014).

So far, it is unclear whether ABA positively or negatively regulates flowering time. A possible scenario to explain the discrepancy is that the effect of ABA on the floral transition depends on the place of action: in the SAM, ABA accumulation results in downregulation of *SOC1* and late flowering, whereas in the leaf, the ABA signaling pathway promotes flowering by upregulating *FT* and *TSF* (Riboni et al., 2013). However, further research will be required to precisely determine the mode of action of ABA in the regulation of flowering time.

GA and ABA work together to regulate flowering time. Double mutants with impaired GA and ABA biosynthesis, for instance, *ga1 aba2* mutants, showed an accelerated flowering time phenotype comparing to that of *ga-1* mutants under LD and SD conditions, indicating that the balance of GAs and ABA is important for the timing of the floral transition (Domagalska et al., 2010). Consistent with this finding, recent studies reported antagonistic crosstalk between ABA and GA signaling in Arabidopsis and rice (*Oryza sativa*). For example, Arabidopsis ABI4 promotes ABA synthesis through NCED6 and inhibits growth and the floral transition; ABI4 also promotes GA degradation through activation of *GA2ox7* expression (Shu et al., 2016). By contrast, accumulation of GA inhibits *ABI4* expression and promotes ABA degradation, thus promoting growth and flowering. A similar case was also observed in rice; in transgenic rice overexpressing *OsAP2-39*, which is an APETALA-2-Like transcription factor, ABA accumulated due to the activation of *OsNCED-1* and GA degradation was promoted by *ELONGATED UPPERMOST INTERNODE* (*OsEUI*), which can be directly activated by ABA (Yaish et al., 2010), indicating that AP2 domain-containing transcription factors play a role in ABA and GA antagonism.

In addition, ABA signaling is important during the drought escape response, in which plants accelerate their flowering in a water-limited environment. During drought escape under LD conditions, ABA upregulates the expression of *GI*, *FT*, and *TSF*, and promotes the floral transition. In rice, the early flowering phenotype seen under low to moderate drought stress conditions was dependent (in part) on ABA signaling (Du et al., 2018). In rice, drought stress caused accumulation of ABA, which upregulates *OsTOC1* and downregulates *OsPHYB* and *GRAIN NUMBER, PLANT HEIGHT AND HEADING DATE 7* (*OsGHD7*), thus promoting flowering. The accumulated ABA regulates photoperiodic and light responses in rice, which affects flowering time. Nevertheless, severe drought stress delays flowering under normal photoperiodic conditions, suggesting the existence of an additional mechanism or blockage of the ABA biosynthesis pathway. It would therefore be interesting to further examine the reasons for the different responses to moderate and strong drought stresses in rice and how different photoperiods affect ABA accumulation.

**Figure 4:**
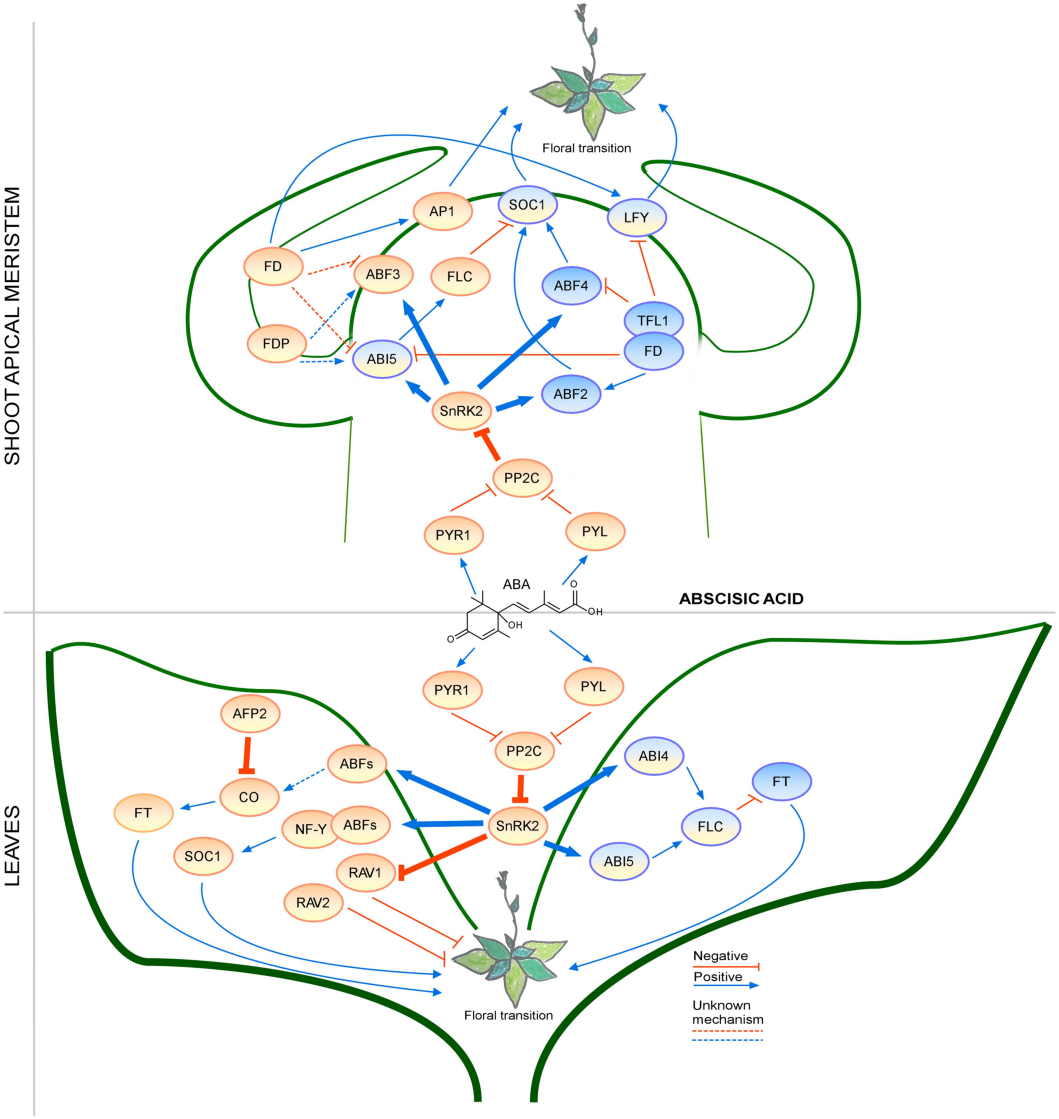
Regulation of the floral transition by the abscisic acid signaling pathway in different photoperiods in SAM and Leaves. Proteins involved in signaling pathways under SD, LD, or both photoperiods are shown in ovals with a blue, orange, or blue-orange background. The positive regulatory interactions are depicted by blue arrows and negative interactions are depicted by red T-bars. The thick lines represent protein-protein interactions, whereas thin lines indicate transcriptional regulation. Unknown mechanisms are depicted by dotted lines. Protein complexes are depicted as partially overlapping ovals.

Abscisic acid signaling during the floral transition has been studied for many years; however, recent findings revealed the presence of additional regulatory mechanisms that require further investigation (Figure 4). For example, the crosstalk with the GA pathway in the regulation of flowering time has emerged as an interesting topic for future studies.

### Cytokinins

Cytokinins (CKs) are synthesized from cytosolic dimethylallyl pyrophosphate and are involved in cell elongation, stress responses, sugar transport, and flowering time regulation (D’Aloia et al., 2011; Kieber and Schaller, 2014). Studies on plant CKs identified crucial proteins involved in CK biosynthesis [ISOPENTENYL TRANSFERASE (IPT)], CK catabolism [CYTOKININ OXIDASE 1 (CKX1) and CKX3], and CK signaling [HISTIDINE KINASE-2 (HK2, HK3, HK4), and ARABIDOPSIS RESPONSE REGULATOR (ARR); D’Agostino et al., 2000; Oka et al., 2002]. Recent studies in Arabidopsis also confirmed that LD photoperiod affects the active transport of cytokinins during the floral transition and CK biosynthesis (Bouché et al., 2016).

Work in the 1960s showed that CK application could induce flowering (Michniewicz and Kamieńska, 1967). The authors showed that treatment with the CK kinetin promotes the floral transition under non-inductive growth conditions in the cold-requiring plant *Cichorium intybus* as well as in the long-day plant *Arabidopsis thaliana*, independently of GA, as endogenous GA levels decrease after kinetin treatment. However, if the CK treatment was performed during early vegetative stages, the treatment delayed flowering rather than inducing flowering (Besnard-Wibaut, 1981). These results showed that CK regulation may lead to opposite outcomes at different developmental stages.

About six decades later, studies confirmed that CKs also act as a flowering time regulator in perennial plants like trees, as apple trees (*Malus domestica*) treated with a synthetic CK showed an accelerated flowering phenotype, together with increased levels of sugars in cytokinin-treated buds (Li et al., 2019b). This finding revealed the relationship between CK signaling and sugar biosynthesis during the floral transition.

Recent studies revealed new roles of the CK sensors in flowering time, based on the characterization of two constitutively active gain-of-function variants of *HK*, named *repressor of cytokinin deficiency* (*rock*; Bartrina et al., 2017). The authors found that introducing *rock2 (HK2^L552F^)* and *rock3 (HK3^T179I^)*, two dominant gain-of-function alleles of *HK2* and *HK3*, respectively, into plants overexpressing the CK catabolic gene *CKX1* rescued the CK-deficiency phenotype (low level of cytokinins and late flowering) under LD conditions, while the high *CKX1* levels and low CK levels were still observed. However, only the *rock2* mutation rescued the non-flowering defect of plants overexpressing *CKX1* under SD conditions, which indicated that the modulation of CK signals acts depending on the photoperiod.

Studies in rice provided new insight into CK signal transmission from HK *via* ARRs (Cho et al., 2016). EARLY HEADING DATE 1 (EHD1), a rice homolog of type-B ARR from Arabidopsis, is a positive regulator of flowering time (Cho et al., 2016). EHD1 forms a homodimer to promote flowering, but heterodimerization of EHD1 with the type-A ARR OsRR1 decreases its ability to promote flowering. Moreover, this regulation was photoperiod-sensitive, as stronger acceleration of flowering was observed in rice *EHD1*-overexpressing plants under LD conditions.

Additionally, recent data revealed that the formation of the TFL1-FD complex leads to downregulation of genes involved in CK biosynthesis and CK signaling (Zhu et al., 2020). TFL1 competes with FT to form a complex with FD to regulates *LFY* expression to control floral induction in the SAM (Zhu et al., 2020). A recent study in barley (*Hordeum vulgare*) proposed that in the photoperiod response, induction of CK biosynthesis and CK signaling are regulated by *CENTRORADIALIS* (*HvCEN*), a homolog of Arabidopsis *TFL1* (Bi et al., 2019). Mutation in *HvCEN* accelerated flowering only under LD conditions, which revealed that CK responses are affected by photoperiod.

Interestingly, CKs may also regulate plant development in coordination with other hormones. For example, *GATA21* and *GATA22* transcription factors, which are involved in light sensing and chloroplast biogenesis, also affect flowering time by repressing *SOC1* expression and are upregulated by CK (Ranftl et al., 2016). The expression of *GATA21* and *GATA22* transcription factors can be controlled by DELLA as well (Richter et al., 2010), which suggests crosstalk among GAs, CKs, and light-sensing pathways in the regulation of flowering time in Arabidopsis. Consistent with this notion, DELLA proteins (GAI and RGA1) were reported to function as co-activators of the CK signaling pathway through the interaction with ARR1 in Arabidopsis (Marín-de la Rosa et al., 2015).

**Figure 5:**
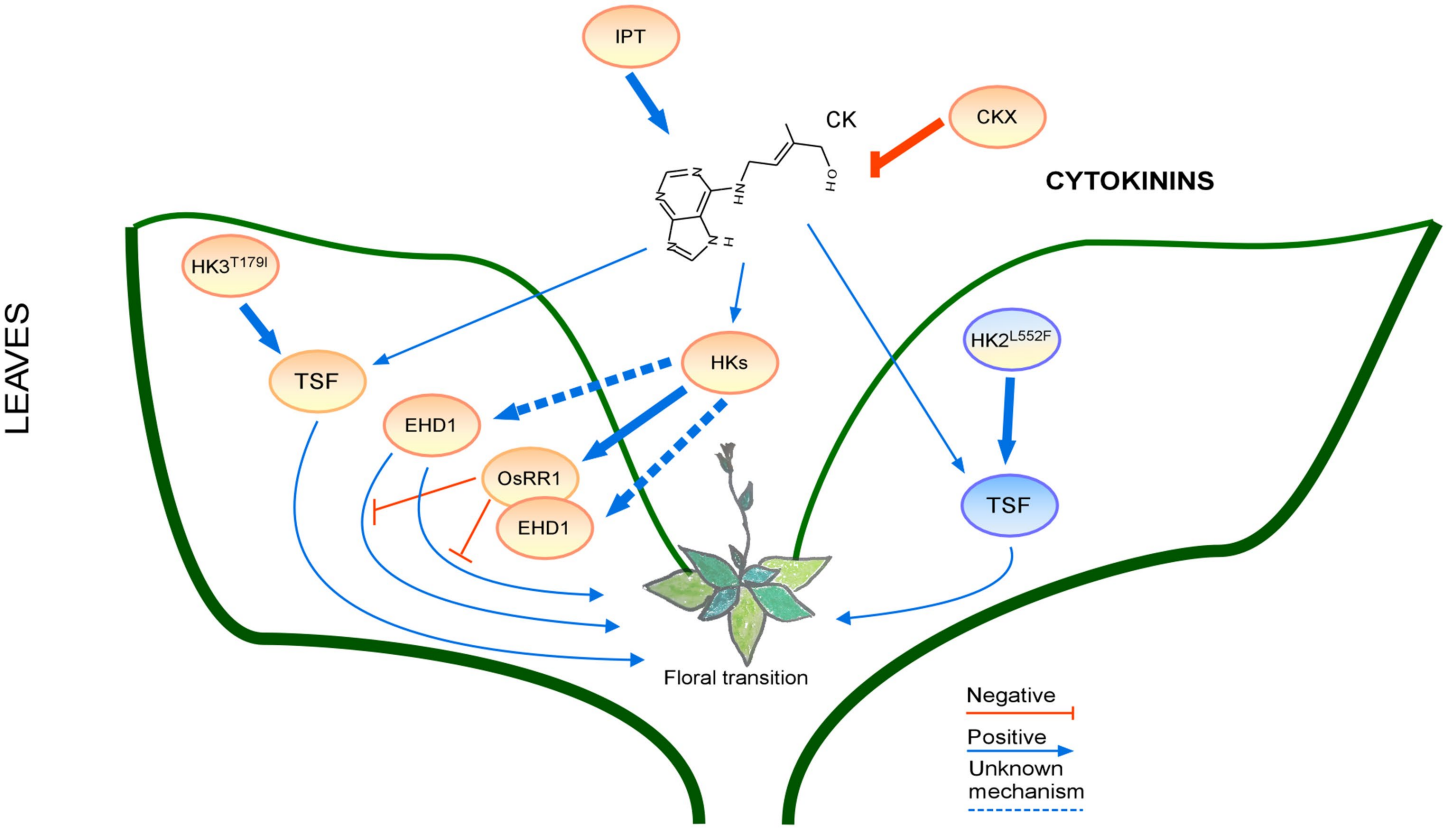
Regulation of the floral transition by the cytokinin signaling pathway in different photoperiods in Leaves. Proteins involved in signaling pathways under SD, LD or both photoperiods are shown in ovals with a blue, orange, or blue-orange background. The positive regulatory interactions are depicted by blue arrows and negative interactions are depicted by red T-bars. The thick lines represent protein-protein interactions, whereas thin lines indicate transcriptional regulation. Protein complexes are depicted as partially overlapping ovals.

The CK signaling network is very complex (Figure 5) and high or low CK levels cause a strong dwarf phenotype with early and late flowering times, respectively. Regulation of CK biosynthesis by many factors involved in flowering time control or light conditions along with crosstalk with other phytohormones make CKs important molecules in plant development. There are still a number of unsolved questions about the cooperation between GAs and CKs and the possibility of other common regulators.

### Photosynthetic Pigments (Carotenoids and Chlorophylls)

Chlorophylls and carotenoids are photosynthetic pigments synthesized from the precursors of the MEP pathway. The levels of these photosynthetic pigments change during the floral transition (Vanacker et al., 2006). These pigments absorb light energy, which is used later to generate fixed carbon sources and light induces the biosynthesis of photosynthetic pigments (Stirbet et al., 2020). Their crucial roles in plant development and abiotic stress responses make them important elements of the flowering time network.

There is no direct evidence of a relationship between chlorophyll accumulation and flowering time; however, studies on plants with altered chlorophyll metabolism showed that flowering time is changed compared to wild-type plants (Table 1). In plants with varied chlorophyll contents, the light signaling and aging pathway in the regulation of flowering time are affected. For example, heterologous overexpression of sweet potato (*Ipomoea batatas*) *VACUOLAR PROCESSING ENZYME 1* (*lbVPE1*), encoding a cysteine proteinase that is involved in the processing of vacuolar proteins and the maturation of seed storage proteins, in Arabidopsis produced an early flowering phenotype under LD conditions and affected chlorophyll catabolism (Jiang et al., 2019). The *IbVPE1-*ovexpressing lines showed accelerated leaf senescence with increased degradation of chlorophyll in the darkness. Furthermore, *IbVPE1*-overexpressing lines had low photosystem II activities and increased *AP1* and *LFY* expression levels. Although there are many mutants affecting chlorophyll metabolism, most of them have not been examined for an effect on flowering time.

**Table 1:** Relationship between chlorophyll content and the regulation of flowering time in response to different photoperiods.

Gene	Species	Effect	Photoperiod conditions	Flowering phenotype	References
**Low chlorophyll levels**					
Heterologous overexpression of *IbVPE1*	Sweet potato	Upregulation of *AP1, LFY*	LD	Early flowering	Jiang et al., 2019
*cry1a/cry2*	Tomato	Downregulation of *SP5G*	LD	Early flowering	Fantini et al., 2019
*ye1*	Rice	Downregulation of *DTH8* (LD and SD) Upregulation of *EF7* (LD)	Photoperiod-insensitive	Early flowering	Peng et al., 2019
**High chlorophyll levels**					
Overexpression of *MtRAV3*	*Medicago truncatula*	Downregulation of *MtFTa1*, *MtSOC1*, and GA biosynthesis	LD	Late flowering	Wang et al., 2021a

Photoreceptors also affect flowering time and chlorophyll accumulation. In tomato, *cry1a cry2* double mutation resulted in a reduction in chlorophyll levels and an early flowering phenotype (Fantini et al., 2019). Tomato *cry1a cry2* double mutants produced fewer leaves than wild type at different light intensities under LD conditions. Subsequent genetic experiments showed that *SELF-PRUNING 5G* (*SP5G*; Cao et al., 2015), a floral inhibitor, was downregulated in *cry1a cry2* mutants under LD conditions, suggesting that SP5G likely promotes flowering in *cry1a cry2* mutants. Studies using rice revealed that a lesion in *YELLOW LEAF AND EARLY FLOWERING* (*YE1*), which encodes a heme oxygenase involved in biosynthesis of the chromatophore of phytochromes, resulted in a reduction in chlorophyll levels and a photoperiod-insensitive early flowering phenotype. Expression analyses using *ye1* mutants revealed altered transcript levels of several genes that are involved in the photoperiod pathway. For instance, the mRNA levels of *EARLY FLOWERING 7* (*EF7*), a rice ortholog of Arabidopsis *ELF3*, which promotes the floral transition both under LD and SD conditions, were significantly higher than wild type in *ye1* mutants under LD conditions, but not under SD conditions (Peng et al., 2019). The expression levels of *DAY TO HEADING 8* (*DTH8*), which encodes a floral repressor and inhibits the expression of florigen under LD conditions, largely decreased, suggesting that *YE1* may control the photoperiodic flowering time by the regulation of the expression of the photoperiodic pathway genes.

By contrast, *Medicago truncatula* plants overexpressing *MtRAV3*, which encodes an AP2/ERF transcription factor, had higher chlorophyll contents compared with wild type and developmental defects including dwarfness and late flowering (Wang et al., 2021a). *MtRAV3-*overexpressing lines showed higher resistance to abiotic stresses under LD conditions and downregulation of *MtFTa1* and *MtSOC1*, along with genes involved in the regulation pathways of GAs and strigolactones; however, the detailed mechanism underlying the observed late-flowering phenotype remains to be examined.

The complex pathways involved in regulating chlorophyll biosynthesis and breakdown, and in leaf senescence may interact with the pathways regulating flowering. However, as chlorophyll contents affect sugar biosynthesis (Yang et al., 2013), additional research will be required to disentangle the effects of chlorophyll and sugars in the regulation of the floral transition.

## Sugar Signaling in Flowering and the Effect of Photoperiod

Sugars are the final products of photosynthesis and are used as a carbon source during the plant’s life cycle; moreover, they serve as important signaling molecules to help plants acclimate to environmental changes and proceed through development (Wingler, 2018). In particular, sugars are important in the transition from the juvenile/vegetative phase to the reproductive phase; here, we will mainly focus on the role of sugars in the regulation of flowering time.

The signals from carbohydrates may differ depending on photoperiodic conditions. For example, after exposure to light, sucrose accumulation in the phloem increased during floral induction in *Sinapis alba* (Lejeune et al., 1993). Starch metabolism was differentially regulated during the floral transition in response to photoperiods and a disturbance in starch metabolism caused a change in flowering time (Ral et al., 2006; Ortiz-Marchena et al., 2014, 2015). CO may play a crucial role in the balance between free sugars and starch during developmental transition from the vegetative to reproductive growth by controlling the timing and the expression levels of *GRANULE BOUND STARCH SYNTHASE* (*GBSS*), which encodes an enzyme that produces linear amylose (Merida et al., 1999; Ortiz-Marchena et al., 2015). The *gbs* mutants showed changes in free sugar content and reduced accumulation of transitory starch, which is the product of photosynthesis formed during the day and is utilized at night, before flowering. In addition to the altered starch composition, the *gbs* mutation caused late flowering, whereas *GBSS* overexpression caused early flowering in Arabidopsis (Ortiz-Marchena et al., 2015). However, the late flowering of *gbs* mutants was observed only under LD conditions, but not under SD conditions, when transitory starch is an important source of sucrose. Moreover, when the *gbs* mutation was introduced into *35S::CO* plants, the early flowering phenotype of *35S::CO* plants was remarkably delayed (Ortiz-Marchena et al., 2014). Additionally, the *gbs* mutation further delayed flowering of *co* mutants, which suggests that *GBSS* also has a developmental role independently of CO (Ortiz-Marchena et al., 2015). A previous study on green algae (*Chlamydomonas reinhardtii*) revealed the connection between *CrCO* expression and starch accumulation (Serrano et al., 2009), showing that the photoperiod regulatory module regulating sugar mobilization by GBSS activity is conserved among plant species. These results showed the importance of proper sugar mobilization, which affects *FT* expression through CO regulation, under LD conditions during the floral transition.

In addition, plants misexpressing *FT* in the SAM had an early flowering phenotype under SD conditions and transcriptome analyses showed that monosaccharide transporter genes were upregulated, whereas the genes encoding sugar transporters were downregulated (Duplat-Bermudez et al., 2016). Arabidopsis, a plant with apoplastic transport of photoassimilates, has a higher demand for glucose and fructose than sucrose in the reproductive stage; however, sucrose was needed to form more leaves in wild-type plants. Therefore, the misexpression of *FT* in the SAM during the stage with high demand for hexoses may accelerate plant growth and flowering (Duplat-Bermudez et al., 2016).

A recent study of saffron (*Crocus sativus*) under cold treatment also showed the connection between flowering and sucrose/starch contents (Chen et al., 2021). In the comparison of sucrose and starch contents during floral transition between normal flowering and non-flowering saffron, the significant reduction in sucrose content, but not starch, was observed in the non-flowering buds. However, the sucrose content of flowering buds was higher than in buds in the dormancy stage. Therefore, the authors speculated that starch/sugar interconversion may be related to the flowering phenotype (Chen et al., 2021). Moreover, exposure to different photoperiods changes the sugar content in *Ranunculus asiaticus*, indicating a positive correlation between early flowering and higher accumulation of free sugars (Modarelli et al., 2020).

In addition to sugar accumulation, carbohydrate transport is an important factor during the floral transition. For example, a recent work showed the positive effect of sugar signaling on flowering time (Wang et al., 2020). When *IbSUT4*, a *SUCROSE TRANSPORTER* from sweet potato, was heterologously overexpressed in Arabidopsis, the *IbSUT4-*overexpressing plants showed early flowering under LD conditions with a significantly increased efflux of sucrose and increased *FT* expression levels. The relationship between sugar transport and photoperiod flowering time was also described by functional analysis of *SWEET10*, a sucrose transporter gene in Arabidopsis. *FT* and *SOC1* can activate the expression of *SWEET10* depending on the photoperiod (Andres et al., 2020). *SWEET10-*overexpressing plants flowered earlier than wild type only under LD conditions and showed high expression levels of *FD*, *SPL4*, and *SPL9* at the shoot apex, with low expression of *miR156*. These results showed the importance of sugar transport during the vegetative to reproductive transition in the SAM.

A moderate amount of sugars in the growth medium can accelerate flowering. However, as most studies analyzing the effect of sugars on flowering time are performed in model plants, not much is known about the regulation of flowering time in non-model plants. Nevertheless, a recent study showed that chrysanthemum (*Chrysanthemum morifolium*) *FT* homologs (*CmFTLs*) may regulate the floral transition (Sun et al., 2017). The authors showed that chrysanthemum treated with exogenous sucrose showed the high induction of *CmFTLs* and flowered early under both LD and SD conditions. Furthermore, the heterologous expression of *CmFTL* rescued the late-flowering phenotype of Arabidopsis *ft-10* mutants.

In addition to sucrose, other carbohydrates may also play a role in the floral transition. For example, trehalose-6-phosphate (T6P) content is regulated in plants by T6P synthase (TPS) and T6P phosphatase (TPP) and T6P accumulation is induced by sucrose (Kolbe et al., 2005). T6P is essential for plants, as the *tps1* mutation is embryo-lethal; however, when *TPS1* was expressed under the control of the seed-specific *ABI3* promoter in the *tps1* background (*tps1 ABI3::TPS1* plants) or from a dexamethasone-inducible construct (*tps1-2 GVG:TPS1* plants), the embryo-lethal phenotype was rescued and very late flowering or even no flowering was observed (van Dijken et al., 2004; Gomez et al., 2010). Further understanding of the molecular mechanism of TPS1 and T6P signaling in the regulation of flowering time was established in 2013. Wahl et al. (2013) confirmed that the expression of *FT* and *TSF* was reduced in the *tps1-2 GVG:TPS1* and *35S::amiR-TPS1* plants under LD conditions, indicating that T6P signaling is a crucial factor in the transcriptional regulation of *FT* and *TSF* under inductive photoperiod conditions. On the other hand, *in situ* hybridization assays and misexpression of *TPS1* using the stem cell niche-specific *CLAVATA3* promoter showed that TPS1 and T6P signaling regulates the floral transition by the controlling the transcription level of *SPL3*, *SPL4*, and *SPL5* in the SAM (Wahl et al., 2013). Taken together, these findings demonstrate that T6P signaling plays a role in flowering time in two different tissues, such that in the leaf, TPS1 is responsible for the induction of *FT* and *TSF* in response to photoperiod, whereas the T6P pathway controls the expression of flowering time and flower-patterning genes *via* the age pathway in the SAM, independent of the photoperiod pathway (Wahl et al., 2013).

Genome-wide analyses in apple trees after exogenous sucrose treatment revealed increased levels of *MdTPS* as well as genes regulating flowering, such as *MdSPL*, *MdFT*, *MdCO*, *MdSOC1*, *MdLFY*, and *MdAP1* (Du et al., 2017). Recent studies examined the function of the non-catalytic domain of TPS1 and how TPS1 contributes to T6P-sucrose nexus (Fichtner et al., 2020). Various mutations including domain deletion and point mutations were introduced into *TPS1* and their effects on flowering and T6p-sucrose contents were analyzed in the *tps1-1* mutant background. In particular, the plants expressing TPS1(A119W), which is expected to compromise catalytic activity, never flowered despite their high T6P levels, indicating that the high levels of T6P may not directly correlate with early flowering (Fichtner et al., 2020). TPS1(A119W) showed not only increased T6P contents but also high levels of two unidentified disaccharide-monophosphates. Therefore, flowering time is probably inhibited by other products that compete with T6P, demonstrating that additional factors that regulate TPS1 activity and affect sugar signaling pathways may exist.

T6P accumulation in plants is negatively regulated by TPP and low T6P positively regulates sugar synthesis. Overexpression of rice *TPP* resulted in reduced T6P levels and increased sugar accumulation in florets in maize, which eventually resulted in increased yields in comparison to wild-type plants (Oszvald et al., 2018). Interestingly, heterologous overexpression of the *Jatropha curcas TPP* gene *JcTPPJ* in Arabidopsis strongly delayed flowering with the accumulation of soluble sugars (Zhao et al., 2019), although its overexpression in Jatropha plants did not change flowering time. These results suggest that T6P degradation is conserved in the plant kingdom but may differ somewhat among plants. Further investigation is needed to elucidate the precise molecular mechanisms in diverse plants.

Sucrose and T6P contents may negatively affect the expression of *SUCROSE-NON-FERMENTING KINASE 1* (*SnRK1*; Baena-Gonzalez et al., 2007; Zhang et al., 2009). It has been proposed that SnRK1 and its substrate INDETERMINATE DOMAIN 8 (IDD8) form a sugar metabolic pathway that mediates flowering time under sugar deprivation conditions. Jeong et al. (2015) showed that phosphorylation of IDD8 by SnRK1 decreased the activity of IDD8 as a transcriptional activator, which altered the expression levels of its downstream genes. The *idd8* mutants show late flowering under LD conditions. As SnRK1 is activated under starvation conditions, it is not surprising that plants overexpressing *AKIN10*, which encodes a catalytic subunit of the SnRK1 complex, and *idd8* mutants show a similar flowering phenotype. Thus, it seems likely that the SnRK1 pathway integrates the metabolic signals into the IDD8-mediated regulatory network. As AKIN10 positively regulates the protein stability of FUSCA3 (FUS3) by phosphorylation in the floral transition (Tsai and Gazzarrini, 2012), it is likely that FUS3 may regulate the floral transition *via* the interaction with IDD8; however, this hypothesis remains to be examined.

**Figure 6:**
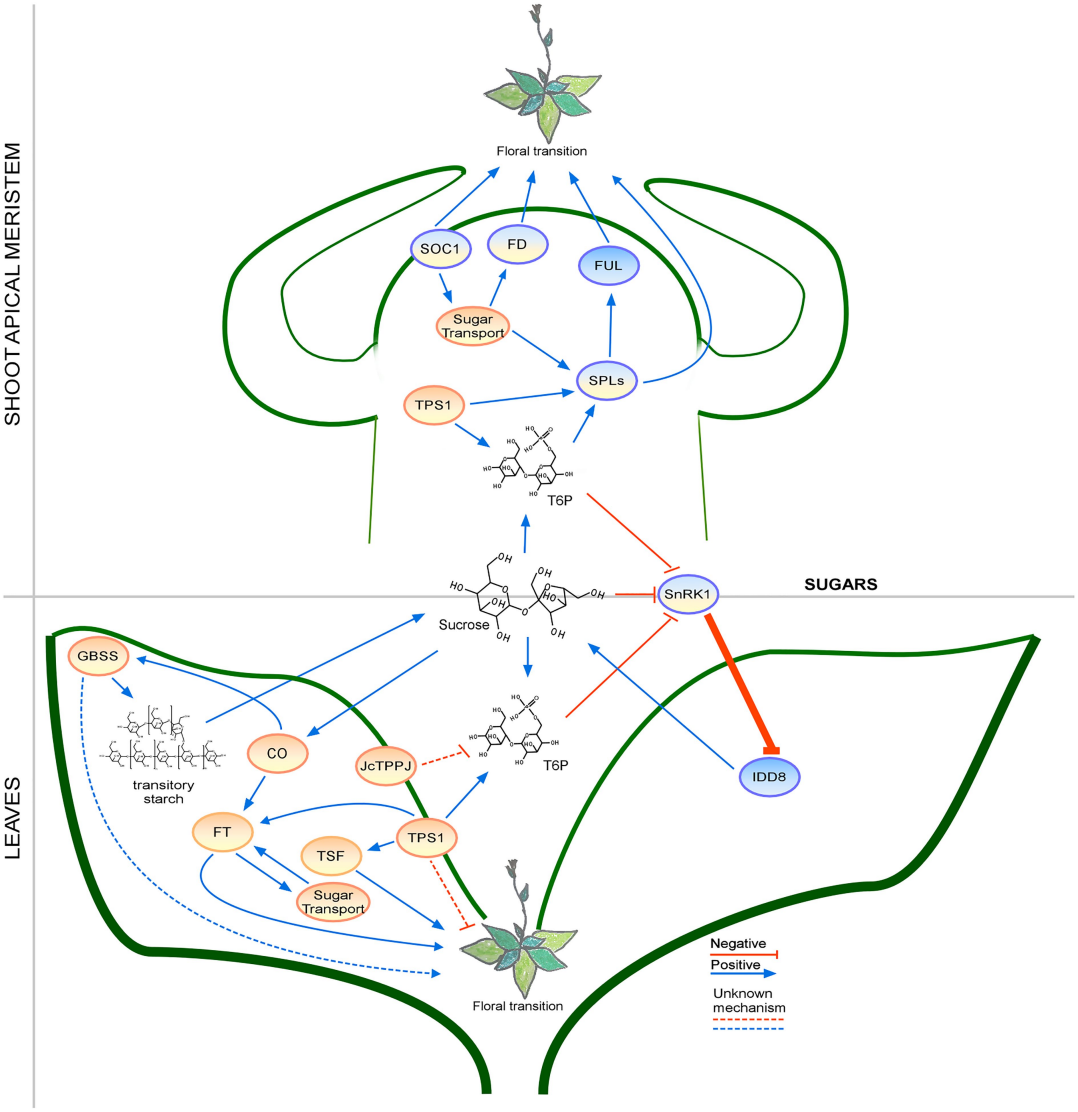
Regulation of the floral transition by the sugar signaling pathway in different photoperiods in SAM and Leaves. Proteins involved in signaling pathways under SD, LD, or both photoperiods are shown in ovals with a blue, orange, or blue-orange background. The positive regulatory interactions are depicted by blue arrows and negative interactions are depicted by red T-bars. The thick lines represent protein-protein interactions, whereas thin lines indicate transcriptional regulation. Unknown mechanisms are depicted by dotted lines and sugar transport is depicted by wavy lines.

Sugar signaling plays an important role during the floral transition and can be regulated by photoperiodic conditions (Figure 6). Although some information on the effects of carbohydrates on flowering time is available, the influence of phytohormones, phosphorylation, and carbohydrates on carbohydrate signaling pathway needs further investigation. In addition, the function of the other two TPSs in Arabidopsis remains to be elucidated (Delorge et al., 2015). Collectively, little is known about carbohydrate signaling during the floral transition and thus it awaits further study.

## Concluding Remarks

Deciding when to flower is a crucial step in the plant life cycle. Successful reproduction and acclimation to the ever-changing environment require the plant to properly sense environmental conditions and its internal status. Plants have established a complicated regulatory network to choose the right timing for the reproductive transition. In this review, we summarized recent findings on the flowering regulators that share isopentenyl diphosphate as a common precursor, as well as sugars, which contribute to some common signaling pathways with specific terpenoids. We focused on the findings that explain how isoprenoid derivatives and sugars regulate flowering time in response to different day-to-night ratios.

The photoperiod affects phytohormones, photosynthetic pigments, and sugars and these signaling pathways eventually modulate the floral transition by modifying the expression of FTGs in the SAM or leaves. As all terpenes share parts of the same biosynthetic pathway, it is not surprising that crosstalk among all phytohormones occurs. Interestingly, depending on the environmental conditions and developmental stages, the interactions among phytohormones, photosynthetic pigments, and sugars can be synergistic or antagonistic (Table 2). Although sugars and terpenoids do not share a biosynthetic pathway, flowering regulation by phytohormones or photosynthetic pigments influences sugar distribution and accumulation. This interaction was also modified in response to photoperiodic conditions. The signals from phytohormones and sugars affect a wide spectrum of flowering activators and repressors, suggesting that phytohormones and sugars are important targets for future research in the study of flowering time.

**Table 2:** A simplified presentation of the crosstalk between different phytohormones and regulation of flowering time in response to photoperiod.

Interacting phytohormones	Organism	Type of interaction	Phytohormone regulation	Flowering phenotype	Photoperiod conditions	References
GA, ABA	Arabidopsis	Antagonistic	Low GA, Low ABA	Rescue of the late-flowering phenotype of *ga-1* by *aba2*	LD and SD	Domagalska et al., 2010
GA, ABA	Arabidopsis	Antagonistic	Low GA, High ABA	Late flowering: Upregulation of *GAox7* by *NCED6*	LD	Shu et al., 2016
GA, ABA	Arabidopsis	Antagonistic	High GA, Low ABA	Early flowering: Inhibition of *ABI4* by GA hormones	LD	Shu et al., 2016
GA, ABA	Rice	Antagonistic	Low GA, High ABA	Late flowering: Upregulation of *OsEUI* by *OsAP2*	LD	Yaish et al., 2010
GA, ABA	Rice	Antagonistic	High GA, Low ABA	Early flowering: Inhibition of *NCED1* by GA hormones	LD	Yaish et al., 2010
CK, GA	Arabidopsis	Synergistic	High CK, High GA	Late flowering: downregulation of *SOC1*	LD	Richter et al., 2010; Ranftl et al., 2016
GA, BR	Arabidopsis	Partially synergistic	High GA, Low BR	Activation of GA synthesis rescued the late-flowering phenotype of *bri-1* mutants	LD	Domagalska et al., 2010; Unterholzner et al., 2015
BR, ABA	Arabidopsis	Antagonistic	High ABA, Low BR	Late flowering: ABA inhibits BR synthesis by inhibition of the PIF − BES1 complex	LD	Hayes et al., 2019

Although past studies showed how phytohormones and sugars are involved in modulating flowering time in response to light, temperature, day length, and stress, recent studies revealed that we are still far from our goal of understanding their molecular mechanisms in the regulation of flowering time. Discoveries of new regulators of terpenes or sugar biosynthesis, as well as factors involved in their sensing and transport, show that the control of flowering time still has unrevealed secrets, especially regarding the points of crosstalk between pathways. Additionally, the effect of phytohormones and carbohydrates on development may differ between plant species; therefore, a better understanding of this regulation in crop species would help improve yields.

Of all the plant phytohormones, it seems that signaling by BRs is less well understood, as BRs were not considered to be involved in the regulation of flowering time until recently. Similarly, not much is known about the regulation of flowering by miRNAs in sugar signaling, as new genes modulated by these factors have been recently discovered in plants. Finally, the identification of new flowering time regulators, such as phospholipids (Susila et al., 2021a,b) and tocopherols (Simancas and Munné-Bosch 2015), has opened new avenues of research into the regulation of flowering time.

## Author Contributions

KG wrote the draft of the manuscript. KG and JHA were involved in editing text and figures. All authors contributed to the article and approved the submitted version.

## Funding

This work was supported by a Korea University Grant, a National Research Foundation (NRF) of Korea grant funded by the Korean Government (NRF-2017R1A2B3009624 to JHA), and Samsung Science and Technology Foundation under Project Number SSTF-BA1602-12 (to JHA).

## Conflict of Interest

The authors declare that the research was conducted in the absence of any commercial or financial relationships that could be construed as a potential conflict of interest.

## Publisher’s Note

All claims expressed in this article are solely those of the authors and do not necessarily represent those of their affiliated organizations, or those of the publisher, the editors and the reviewers. Any product that may be evaluated in this article, or claim that may be made by its manufacturer, is not guaranteed or endorsed by the publisher.
